# 131. Antiviral NL-CVX1 Efficiently Blocks Infection of SARS-CoV-2 Viral Variants of Concern (VOC)

**DOI:** 10.1093/ofid/ofab466.131

**Published:** 2021-12-04

**Authors:** Wen Su, Matthew Walker, Maria Rebelo, Cong Tang, Ana R Coelho, Laurie Tatalick, Marianne Riley, Kevin Yu, Luis M Blancas-Mejia, Daniel-Adriano Silva, David Shoultz, Goncalo Bernardes, Hui-Ling Yen

**Affiliations:** 1 The University of Hong Kong, Pokfulam, Not Applicable, Hong Kong; 2 Neoleukin Therapeutics, Inc., Seattle, Washington; 3 Institute for Molecular Medicine, University of Lisbon, Lisbon, Lisboa, Portugal; 4 University of Cambridge, Cambridge, England, United Kingdom; 5 Laurie Tatalick Consulting, Richmond, Virginia

## Abstract

**Background:**

Using a computational approach, NL-CVX1 was developed by Neoleukin Therapeutics, Inc. to create a *de novo* protein that both blocks SARS-CoV-2 infection and is highly resilient to viral escape. In this study we evaluated the efficacy of NL-CVX1 against variants of the original SARS-CoV-2 strain, including important viral variants of concern (VOC) such as B.1.1.7, B.1.351, and P.1.

**Methods:**

The relative binding affinity of NL-CVX1 to the SARS-CoV-2 viral spike protein of VOC was measured using biolayer interferometry (Octet). A competitive ELISA measured the ability of NL-CVX1 to compete with hACE2 for binding to the receptor binding domain (RBD) of the SARS-CoV-2 S protein from the original strain and VOC. The activity of NL-CVX1 in preventing viral infection was assessed by evaluating the cytopathic effects (CPE) of SARS-CoV-2 in a transmembrane protease, serine 2-expressing Vero E6 cell line (Vero E6/TMPRSS2) and determining the viral load using quantitative real-time reverse transcriptase polymerase chain reaction in infected cells. A K18hACE2 mouse model of SARS CoV-2 infection was used to study the dose-response of NL-CVX1 anti-viral activity *in vivo*.

**Results:**

NL-CVX1 binds the RBD of different VOC of SARS-CoV-2 at low nanomolar concentrations (Fig 1; K_d_ < 1-~5 nM). When competing with hACE2, NL-CVX1 achieved 100% inhibition against hACE2 binding to the RBD of different VOC with IC50s values ranging from 0.7-53 nM (Fig 2). NL-CVX1 neutralized the B.1.1.7 variant as efficiently as the original strain in Vero E6/TMPRSS2 cells, with EC50 values of 16 nM and 101.2 nM, respectively (Fig 3). In mice, we found that a single intranasal dose of 100 µg NL-CVX1 prevented clinically significant SARS-CoV-2 infection and protected mice from succumbing to infection. Results from additional *in vitro* and *in vivo* experiments to be conducted this summer will be presented.

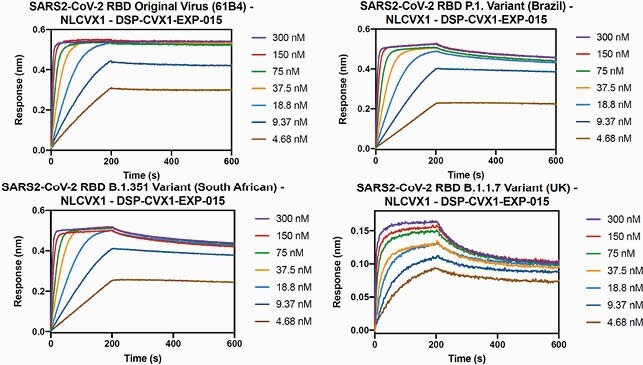

Figure 1. NL-CVX1 binds the RBD from multiple strains of SARS-CoV-2 at low nanomolar concentrations.

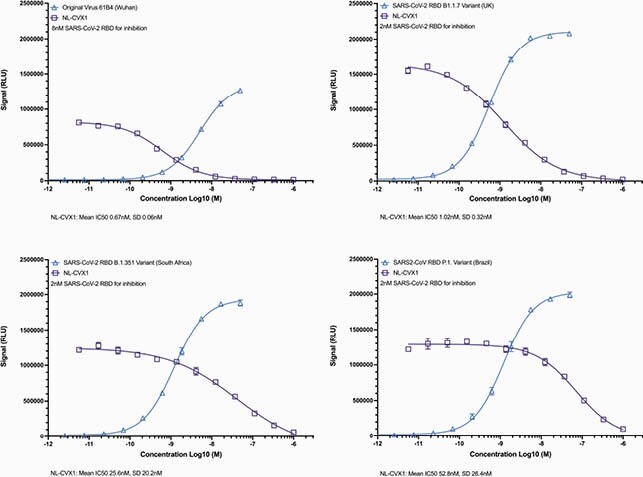

Figure 2. NL-CVX1 achieves 100% inhibition against all strains tested, including SARS-CoV-2 VOC.

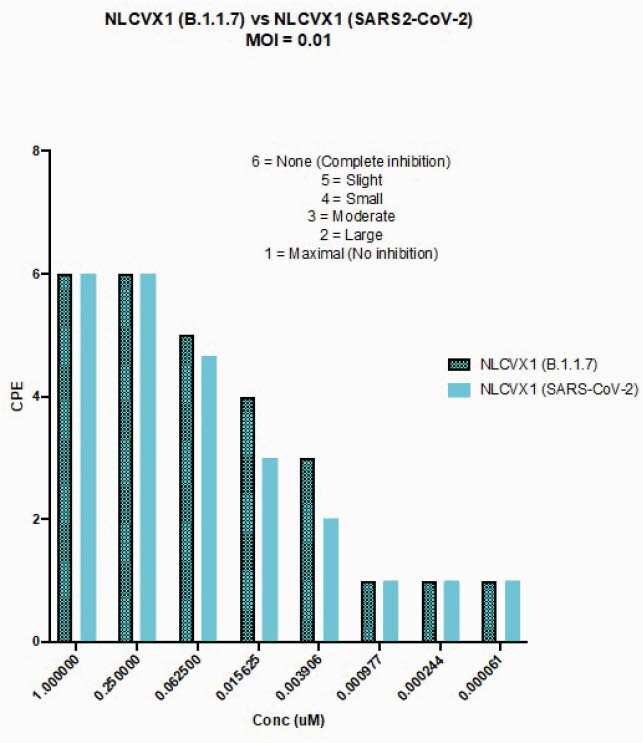

Figure 3. NL-CVX1 neutralizes the B.1.1.7 variant as efficiently as the original SARS-CoV-2 strain.

**Conclusion:**

*In vitro* and *in vivo* data (Fig 4) demonstrate that NL-CVX1 is a promising drug candidate for the prevention and treatment of COVID-19. As a hACE2 mimetic, it is resilient to antibody escape mutations found in SARS-CoV-2 VOC. NL-CVX1 further demonstrates the power and utility of *de novo* protein design for developing proteins as human therapeutics.

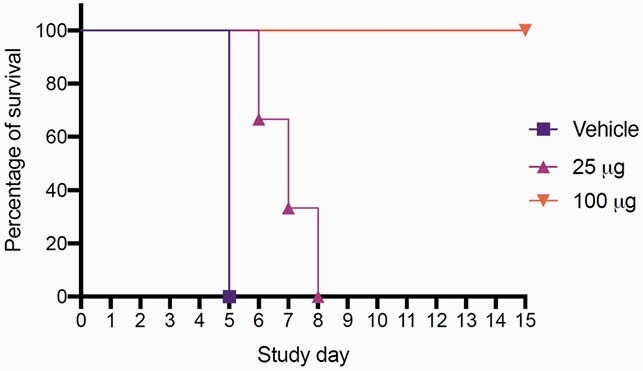

Figure 4. NL-CVX1 is effective in preventing clinically significant SARS-CoV-2 viral infection in a K18hACE2 mouse model.

**Disclosures:**

**Matthew Walker, PhD**, **Neoleukin Therapeutics, Inc.** (Employee, Other Financial or Material Support, Ownership options and stock.) **Laurie Tatalick, DVM, PhD, DACVP**, **Neoleukin Therapeutics, Inc.** (Consultant, Other Financial or Material Support, Ownership options and stock.) **Marianne Riley, BS**, **Neoleukin Therapeutics, Inc.** (Employee, Other Financial or Material Support, Ownership options and stock.) **Kevin Yu, BS, MS**, **Neoleukin Therapeutics, Inc.** (Employee, Other Financial or Material Support, Ownership options and stock.) **Luis M. Blancas-Mejia, PhD**, **Neoleukin Therapeutics, Inc.** (Employee, Other Financial or Material Support, Ownership options and stock.) **Daniel-Adriano Silva, PhD**, **Neoleukin Therapeutics, Inc.** (Advisor or Review Panel member, Other Financial or Material Support, Ownership of Neoleukin options and stock) **David Shoultz, PhD, MBA**, **Neoleukin Therapeutics, Inc.** (Employee, Other Financial or Material Support, Ownership options and stock.) **Goncalo Bernardes, PhD**, **Neoleukin Therapeutics, Inc.** (Consultant, Advisor or Review Panel member, Shareholder) **Hui-Ling Yen, PhD**, **Neoleukin Therapeutics, Inc.** (Grant/Research Support)**Saiba AG** (Other Financial or Material Support, Received donation from Saiba AG)

